# Retinal Vascular Morphology Reflects and Predicts Cerebral Small Vessel Disease: Evidences from Eye–Brain Imaging Analysis

**DOI:** 10.34133/research.0633

**Published:** 2025-03-06

**Authors:** Ning Wu, Mingze Xu, Shuohua Chen, Shouling Wu, Jing Li, Ying Hui, Xiaoshuai Li, Zhenchang Wang, Han Lv

**Affiliations:** ^1^ Department of Medical Imaging, Yanjing Medical College, Capital Medical University, Beijing, China.; ^2^ Center for MRI Research, Academy for Advanced Interdisciplinary Studies, Peking University, Beijing, China.; ^3^ Department of Cardiology, Kailuan General Hospital, Tangshan, China.; ^4^ Department of Radiology, Beijing Tsinghua Changgung Hospital, School of Clinical Medicine, Tsinghua University, Beijing, China.; ^5^ Department of Radiology, Kailuan General Hospital, Tangshan, China.; ^6^ Department of Radiology, Capital Medical University Affiliated Beijing Friendship Hospital, Beijing, China.

## Abstract

Cerebral small vessel disease (SVD) involves ischemic white matter damage and choroid plexus (CP) dysfunction for cerebrospinal fluid (CSF) production. Given the vascular and CSF links between the eye and brain, this study explored whether retinal vascular morphology can indicate cerebrovascular injury and CP dysfunction in SVD. We assessed SVD burden using imaging phenotypes like white matter hyperintensities (WMH), perivascular spaces, lacunes, and microbleeds. Cerebrovascular injury was quantified by WMH volume and peak width of skeletonized mean diffusivity (PSMD), while CP volume measured its dysfunction. Retinal vascular markers were derived from fundus images, with associations analyzed using generalized linear models and Pearson correlations. Path analysis quantified contributions of cerebrovascular injury and CP volume to retinal changes. Support vector machine models were developed to predict SVD severity using retinal and demographic data. Among 815 participants, 578 underwent ocular imaging. Increased SVD burden markedly correlated with both cerebral and retinal biomarkers, with retinal alterations equally influenced by cerebrovascular damage and CP enlargement. Machine learning models showed robust predictive power for severe SVD burden (AUC was 0.82), PSMD (0.81), WMH volume (0.77), and CP volume (0.80). These findings suggest that retinal imaging could serve as a cost-effective, noninvasive tool for SVD screening based on vascular and CSF connections.

## Introduction

Cerebral small vessel disease (SVD) is a leading cause of neurological disorders, responsible for approximately 25% of ischemic strokes and 45% of dementia cases [1,2]. Magnetic resonance imaging (MRI) is the gold standard for diagnosing SVD, with white matter hyperintensities (WMHs) as the primary marker. The prevalence of SVD is particularly high among older adults, affecting up to 90% of individuals over the age of 80 [3], and timely diagnosis and intervention may reduce the risk of stroke and dementia [4]. However, due to its high costs, long scan time, and the need for specialized expertise, MRI is limited in its use for large-scale screening.

Humans are complex organisms with coordinated multi-organ systems [5]. The eye provides a unique opportunity to directly observe the body’s microvascular system, making it a valuable “window” into brain health. Fundus photography, being cost-effective and noninvasive, shows great potential as an alternative for diagnosing SVD and related brain pathologies [6]. However, the mechanisms linking retinal vascular changes to cerebral abnormalities in SVD remain unclear. Specifically, it is not yet fully understood which cerebral pathologies can be reflected by retinal imaging.

The retina is an extension of the diencephalon, with a close anatomical relationship and similar vascular regulatory mechanisms shared between the blood supply of both organs [7]. Decreased retinal vascular density (VD) was associated with severe WMH in the brain [8], indicative of macrostructural white matter damage caused by chronic ischemia.

Beyond the vascular connections, researchers have demonstrated cerebrospinal fluid (CSF) circulation around the optic nerve in rodent models using CSF tracers [9], offering new insights into the role of CSF dynamics in ocular diseases. For instance, glaucoma patients exhibit lower intracranial pressure compared to nonglaucoma controls, which is maintained by the balance between CSF production and outflow [10]. The choroid plexus (CP) is a network of blood vessels and epithelial cells located within the brain’s ventricles, which not only produces CSF but also supplies micronutrient, clears the waste, and maintains hemostasis by CSF circulation [11]. CP enlargement has been associated with WMH growth [12] and cognitive decline [13]. However, the relationship between CP dysfunction and retinal vascular morphology remains largely unexplored.

Integrating artificial intelligence with retinal imaging holds the potential to transform the detection and monitoring of SVD, providing a rapid, accessible, and cost-effective solution for large-scale screening and early diagnosis of cerebrovascular disease. Although there have been attempts to utilize deep learning with retinal imaging to predict the severity [14] and locations [15] of WMH, it remains uncertain whether more interpretable retinal vascular features can simultaneously predict SVD burden and its underlying mechanisms, particularly CP dysfunction.

**Fig. 1.**
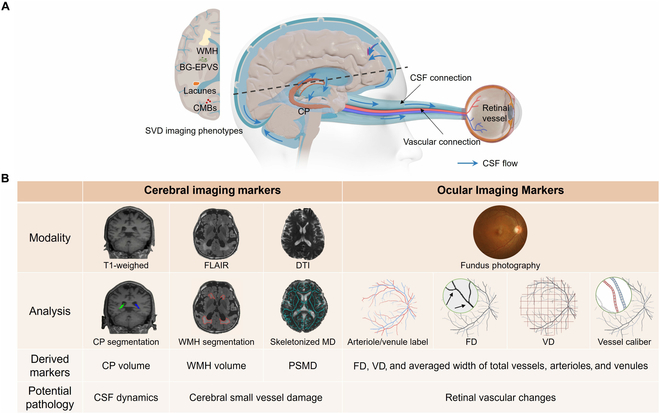
Overview of eye–brain markers and hypothesized pathological connections in this study. (A) Schematic representation of the vascular and cerebrospinal fluid (CSF) connection between the eye and brain. (B) Imaging modalities, analyses, and potential pathologies of the markers in this study. We hypothesize that retinal vascular changes in cerebral small vessel disease (SVD) patients are influenced by cerebrovascular damage and enlarged choroid plexus (CP) through the vascular and CSF connection. SVD burden scores were manually assessed using 4 imaging phenotypes: white matter hyperintensities (WMHs), enlarged perivascular spaces in basal ganglia (BG-EPVS), lacunes, and cerebral microbleeds (CMBs). For cerebral imaging markers, CP volume was segmented from T1-weighted images to reflect CSF dynamics. Both WMH and peak width of skeletonized mean diffusivity (PSMD) serve as markers of SVD driven by chronic ischemia; WMH volume was calculated on fluid-attenuated inversion recovery (FLAIR) images, while PSMD value was derived from skeletonized mean diffusivity in diffusion tensor imaging (DTI). Ocular imaging markers were derived from fundus photography, including artery/vein labeling, and the calculation of fractal dimension (FD), vessel vascular density (VD), and vessel caliber.

In this study, we hypothesize that retinal vascular abnormalities in SVD patients are influenced by, and can predict, both cerebrovascular injury and CP dysfunction, based on the vascular and CSF connections between the eye and brain (illustrated in Fig. 1A). To test this, we compared cerebral and retinal imaging biomarkers (showed in Fig. 1B) across different SVD burden levels, examined correlations between them, quantified the contributions of cerebrovascular injury and CP enlargement to retinal vascular changes, and constructed machine learning models using retinal features and demographic data to predict the severity of SVD and cerebral imaging markers.

## Results

### Participant characteristics

After excluding 299 participants due to insufficient clinical data (*N* = 235) and incomplete MRI scans (*N* = 64), 815 participants (mean age was 56.02 ± 10.85 years, 54% was female) were included in the cerebral imaging analysis, with 578 of them also included in the ocular imaging analysis based on available fundus photography data. The detailed flowchart was depicted in Fig. S1.

Among 815 participants, 254 (31.2%) had an SVD burden score of 0, 258 (31.7%) had a score of 1, 163 (20.0%) had a score of 2, and 140 (17.2%) had a score of 3 or 4 (Table). Higher SVD burden scores were associated with older ages (*F* = 194.20, *P* < 0.001), lower proportions of females (χ^2^ = 66.77, *P* < 0.001), fewer years of education (*F* = 70.79, *P* < 0.001), lower Montreal Cognitive Assessment scores (*H* = 150.28, *P* < 0.001), and higher body mass index (BMI) (*F* = 3.77, *P* = 0.01). Manually assessed SVD markers, including numbers of WMH burden (χ^2^ = 440.55, *P* < 0.001), lacunes (χ^2^ = 442.35, *P* < 0.001), cerebral microbleeds (χ^2^ = 314.96, *P* < 0.001), and enlarged perivascular spaces in basal ganglia (χ^2^ = 571.99, *P* < 0.001) were all significantly increased with SVD burden scores.

**Table.** Clinical characteristics according to cerebral small vessel disease burden scores. Continuous variables are determined with ANOVA or Kruskal–Wallis test as appropriate; Categorical variables are determined with χ^2^ test. BG-EPVS, enlarged perivascular spaces in basal ganglia; BMI, body mass index; CMBs, cerebral microbleeds; MoCA, Montreal Cognitive Assessment; SVD, cerebral small vascular diseases; WMH, white matter hyperintensities.

Characteristics	SVD burden scores				
	0 (*N* = 254)	1 (*N* = 258)	2 (*N* = 163)	≥3 (*N* = 140)	*P* value
Age (years), mean ± SD	47.0 ± 8.1	56.0 ± 8.6	61.3 ± 8.2	66.3 ± 8.0	<0.001
Female, no. (%)	154 (60.6)	127 (49.2)	63 (38.7)	27 (19.3)	<0.001
Education (years), mean ± SD	15.8 ± 2.4	14.1 ± 3.3	12.5 ± 3.3	11.6 ± 3.3	<0.001
MoCA, median (IQR)	27.0 (25–28)	25.0 (23–27)	24.0 (21–27)	22.5 (19–25)	<0.001
Vascular risk factors					
Hypertension, no. (%)	57 (22.4)	85 (32.9)	74 (45.4)	87 (62.1)	<0.001
Diabetes, no. (%)	22 (8.7)	46 (17.8)	42 (25.8)	46 (32.9)	<0.001
Dyslipidemia, no. (%)	154 (60.6)	172 (66.7)	117 (71.8)	88 (62.9)	0.110
BMI (kg/m^2^), mean ± SD	24.8 ± 3.2	25.1 ± 3.2	25.6 ± 3.1	25.8 ± 3.0	0.010
Current drinking, no. (%)	47 (18.5)	69 (26.7)	44 (27.0)	37 (26.4)	0.090
Current smoking, no. (%)	33 (13.0)	42 (16.3)	43 (26.4)	31 (22.1)	0.003
SVD markers, no. (%)					
High WMH burden	0 (0)	22 (8.5)	82 (50.3)	123 (87.9)	<0.001
Prescence of lacunes	0 (0)	2 (0.8)	26 (16.0)	104 (74.3)	<0.001
Prescence of CMBs	1 (0.4)	40 (15.5)	65 (39.9)	111 (79.3)	<0.001
Moderate to severe BG-EPVS	2 (0.8)	195 (75.6)	155 (95.1)	139 (99.3)	<0.001

### Cerebral and retinal imaging markers varied across SVD burdens

**Fig. 2.**
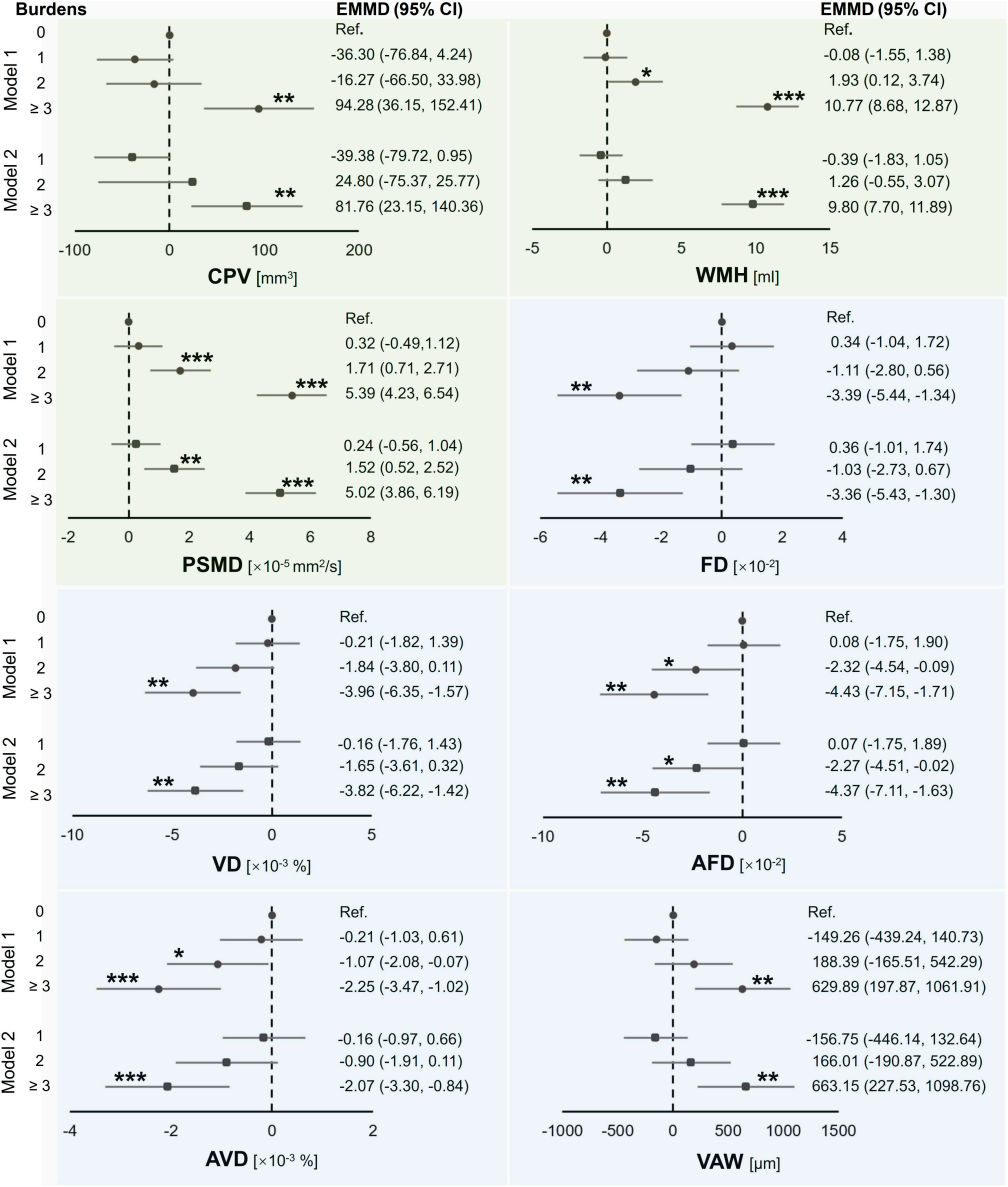
Changes in cerebral and retinal imaging markers across different SVD burden levels compared to burden 0. Generalized linear models were used, adjusting for age, sex, and years of education in model 1, with additional adjustments for hypertension, diabetes, dyslipidemia, body mass index (BMI), and current smoking and drinking status in model 2. Markers on the green background were derived from cerebral imaging, and those on the blue background were from retinal imaging. CPV, choroid plexus volume; EMMD, estimated marginal mean difference; AFD, arteriolar FD; AVD, arteriolar VD; VAW, venular averaged width. ***P < 0.001; **P < .01; *P < 0.05.

We assessed the differences in cerebral and retinal imaging biomarkers across varying SVD burden levels compared to individuals with a burden of 0, as shown in Fig. 2. For cerebral imaging biomarker, significant increases were observed in CP volumes (*P* = 0.001 in model 1 and *P* = 0.006 in model 2 at burden 3), WMH volumes (both *P* < 0.001 at burden 3; *P* = 0.037 in model 1 at burden 2), and peak width of skeletonized mean diffusivity (PSMD) values (*P* < 0.001 in model 1 and *P* = 0.03 in model 2 at burden 2; both *P* < 0.001 at burden 3).

For retinal imaging markers, we found significantly decreased fractal dimension (FD; both *P* = 0.001 at burden 3), VD (*P* = 0.001 in model 1 and *P* = 0.002 in model 2 at burden 3), arteriolar FD (*P* = 0.042 in model 1 and *P* = 0.048 in model 2 at burden 2; *P* = 0.001 in model 1 and *P* = 0.002 in model 2 at burden 3), and arteriolar VD (both *P* < 0.001 at burden 3; *P* = 0.036 in model 1 at burden 2), along with an increase in venular averaged width (VAW; *P* = 0.004 in model 1 and *P* = 0.003 in model 2 at burden 3).

### Correlation between cerebral and retinal imaging markers

**Fig. 3.**
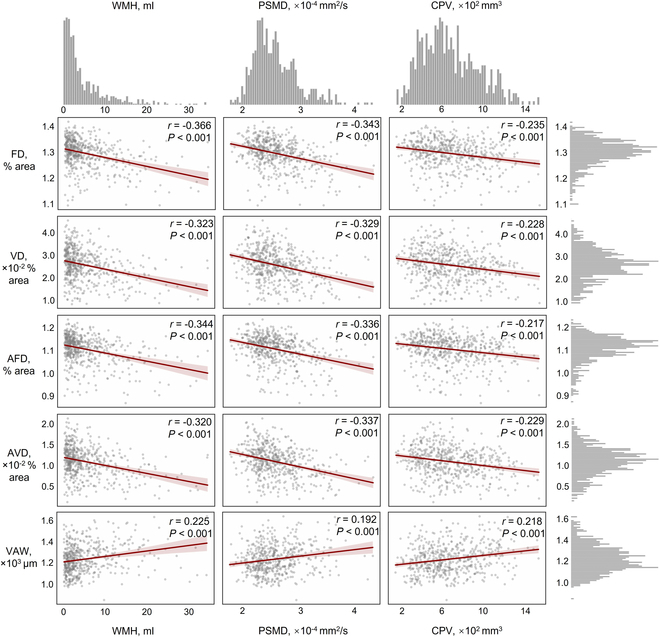
Correlation analysis between cerebral and retinal imaging markers in SVD patients. Scatter plots depict the relationships between cerebral imaging markers (WMH volume, PSMD value, and CPV) and retinal vascular biomarkers (FD, VD, AFD, AVD, and VAW). The top and right margins of the figure show histograms representing the distribution of each imaging marker.

Figure 3 illustrates the significant correlations between cerebral and retinal imaging biomarkers (all *P* < 0.001). WMH volume, PSMD value, and CP volume showed negative correlations with retinal FD, VD, arteriolar FD, and arteriolar VD, and positive correlations with VAW. Specifically, WMH volume correlated with FD (*r* = −0.366), VD (*r* = −0.323), arteriolar FD (*r* = −0.344), arteriolar VD (*r* = −0.320), and VAW (*r* = 0.225). PSMD value showed correlations with FD (*r* = −0.343), VD (*r* = −0.329), arteriolar FD (*r* = −0.336), arteriolar VD (*r* = −0.337), and VAW (*r* = 0.192). CP volume correlated with FD (*r* = −0.235), VD (*r* = −0.228), arteriolar FD (*r* = −0.217), arteriolar VD (*r* = −0.229), and VAW (*r* = 0.218). These findings demonstrate a consistent relationship between retinal vascular morphology and SVD markers or CP volume.

### Partial least squares structural equation modeling

**Fig. 4.**
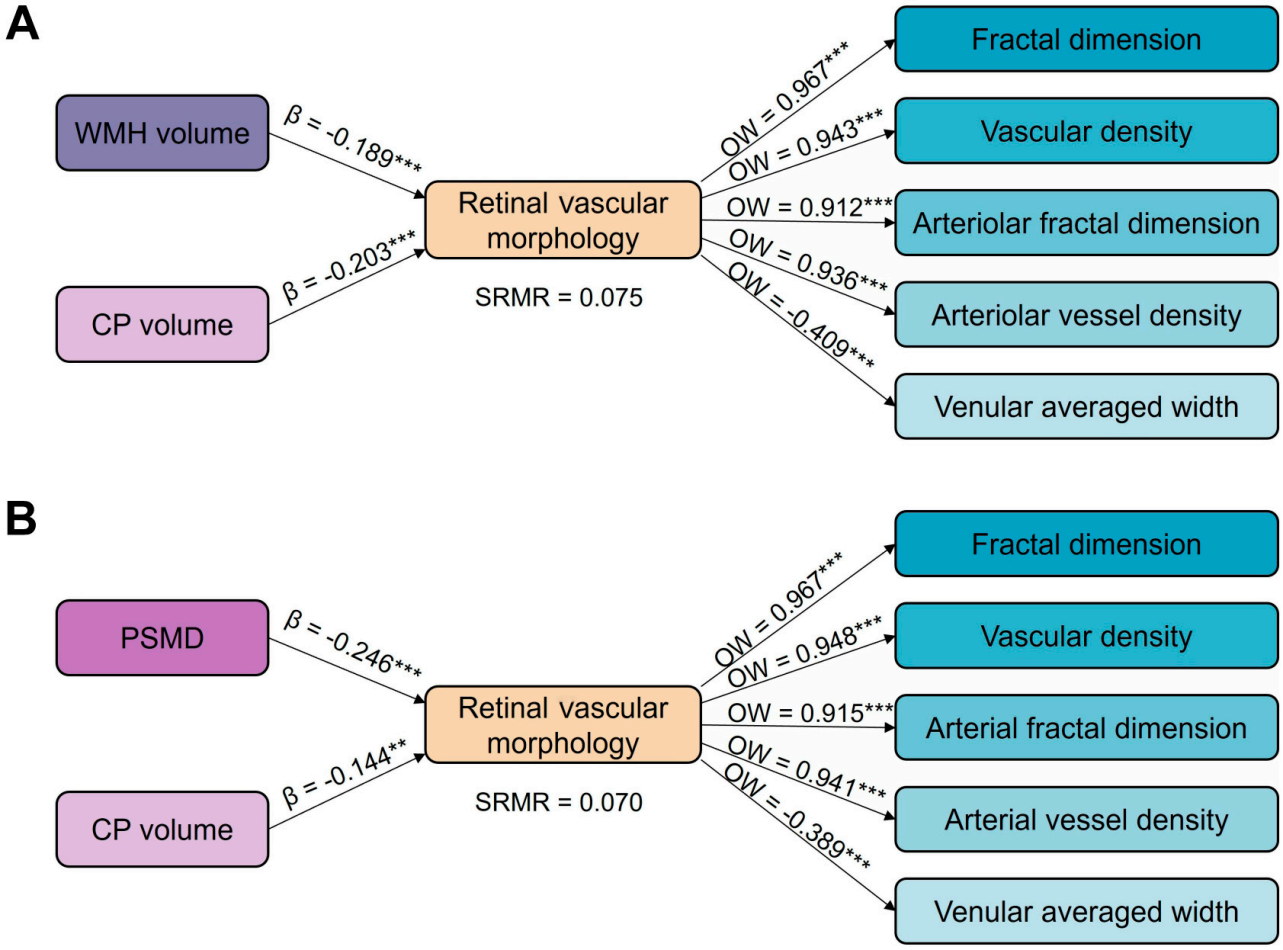
Results of partial least squares structural equation modeling showing the contributions of CP volume and WMH volume (A) or PSMD (B) to retinal vascular morphology. Retinal vascular morphology is the latent variable, formed by observed variables, including FD and VD of total vessel and arterioles, alongside VAW. SRMR (standardized root mean square residual) indicates the difference between observed and predicted correlations. OW (outer weight) indicates the relative contribution of each indicator to its corresponding latent variable, which is retinal vascular morphology.

Figure 4 illustrates the results of partial least squares structural equation modeling, which evaluate the influence of cerebrovascular injury and CP dysfunction on retinal vascular morphology. Two models were constructed based on different cerebrovascular imaging biomarkers (WMH in model A and PSMD in model B). Both models demonstrated a good fit, with standardized root mean square residual values of 0.075 and 0.070, respectively.

In model A, WMH explained 48.2% and CP volume 51.8% of the variance in retinal vascular morphology, respectively. In model B, PSMD explained 63.1% and CP volume 36.9% of the variance in retinal vascular morphology, respectively. All retinal imaging markers showed strong contribution to the variance in retinal vascular morphology (all *P* < 0.001).

### Prediction models and external validation

**Fig. 5.**
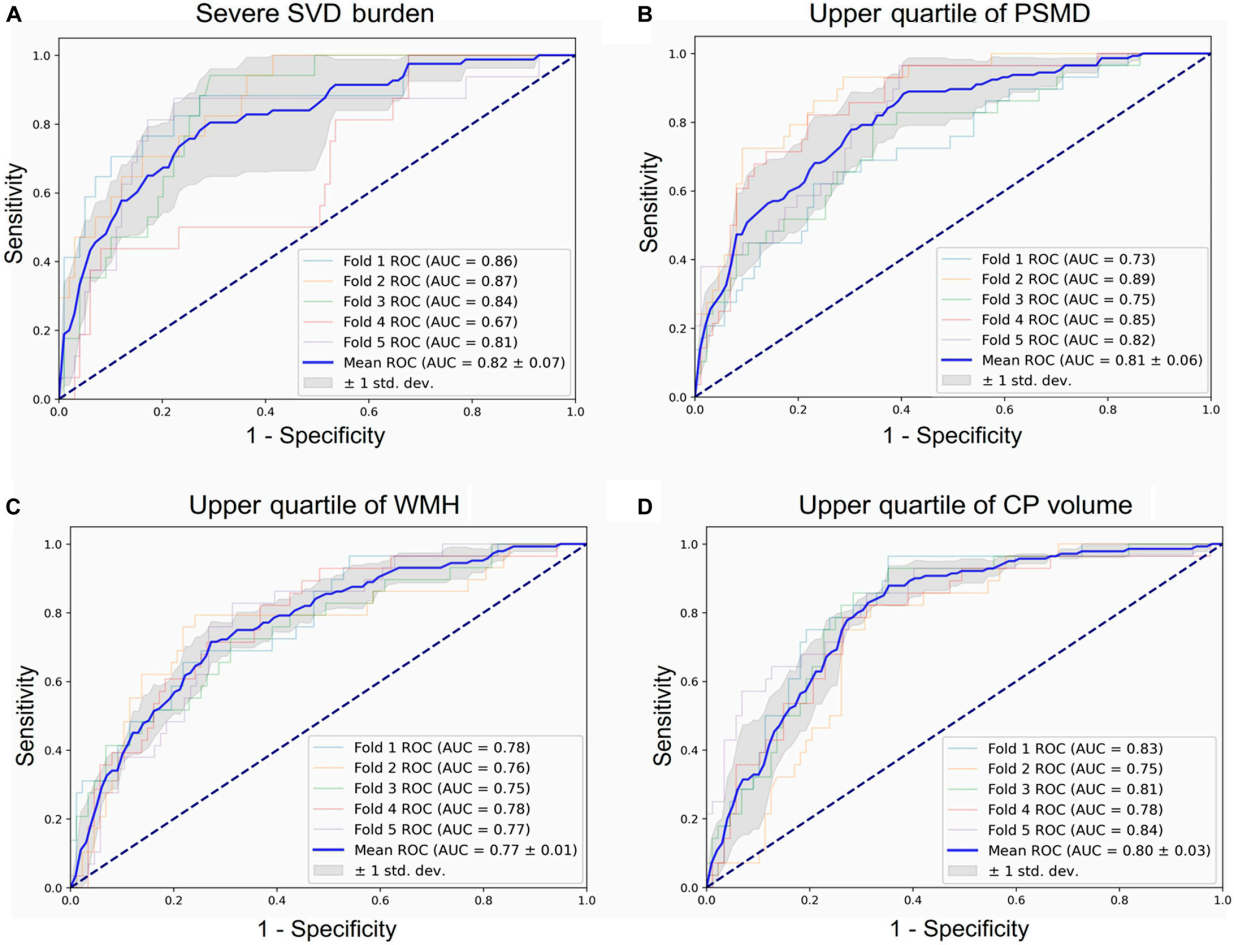
Receiver operating characteristic curves of support vector machine models for predicting severe SVD burden (A), upper quartile of PSMD (B), WMH (C), and CP volume (D). Each panel presents the receiver operating characteristic (ROC) curves for 5-fold cross-validation, with the area under the curve (AUC) values for each fold and their mean value. The shaded area represents the standard deviation of the ROC curves across the 5 folds. Performance metrics, including mean accuracy, sensitivity, and specificity, are displayed below each panel. Severe SVD burden was defined as a burden score of 3 or above.

Figure 5 presents the predictive results and performance parameters of the machine learning models based on 5 retinal imaging biomarkers and demographic data. The highest mean area under the curve (AUC) from the 5-fold cross-validation was observed for the prediction of SVD burden (0.82 ± 0.07), followed by predictions for the upper quartile of PSMD (0.81 ± 0.06), CP volume (0.80 ± 0.03), and WMH (0.77 ± 0.01).

In terms of other model performance, the prediction of SVD burden achieved the highest overall accuracy (0.77 ± 0.05) and specificity (0.78 ± 0.05) while maintaining balanced sensitivity (0.72 ± 0.12). The PSMD and WMH prediction models showed comparable results, with accuracies of 0.71 ± 0.08 and 0.72 ± 0.02, and specificities of 0.71 ± 0.10 and 0.74 ± 0.04, respectively. The model for CP volume achieved the highest sensitivity (0.89 ± 0.05) with relatively lower accuracy (0.67 ± 0.04) and specificity (0.61 ± 0.05).

Additionally, we validated the reliability of the prediction model using the UK Biobank (UKB) dataset, receiver operating characteristic curves of which were provided in Fig. S2. The average AUC for the WMH prediction model was 0.76 ± 0.01, with mean accuracy, specificity, and sensitivity of 0.67 ± 0.02, 0.64 ± 0.02, and 0.75 ± 0.02, respectively. For the CP volume prediction model, the average AUC, accuracy, specificity, and sensitivity were 0.81 ± 0.01, 0.74 ± 0.01, 0.74 ± 0.02, and 0.73 ± 0.02, respectively.

## Discussion

This study provides the first imaging evidence of a CSF connection between the eye and brain, complementing the established vascular link. It further demonstrates that CP volume, responsible for CSF secretion, has an effect on retinal vascular changes comparable to that of cerebral vascular damage biomarkers (including WMH volumes and PSMD values). Additionally, using the same limited retinal imaging biomarkers, lightweight machine learning models were developed to accurately predict SVD score, cerebral vascular biomarkers, and CP volume.

Our study’s findings that WMH volumes and PSMD markedly increased with SVD severity were consistent with prior research, highlighting their roles as markers of cerebrovascular damage. WMH reflected ischemic injury in white matter due to chronic hypoperfusion, leading to demyelination, axonal loss, and gliosis, processes strongly associated with SVD progression and cognitive decline [16]. Meanwhile, PSMD captures microstructural changes in the white matter, which are similarly linked to prolonged ischemic damage from small vessel dysfunction [17] and were closely correlated with WMH score and volume [18].

Impaired CSF waste clearance is a key factor in the development of SVD, as the glymphatic system, responsible for removing metabolic waste from the brain, becomes less effective. This dysfunction leads to the accumulation of harmful proteins like amyloid-β, contributing to neuroinflammation and further vascular damage [19,20]. Our finding of a marked association between increased CP volume and SVD severity supported the role of CSF dysregulation in SVD progression. CP enlargement may reflect compensatory changes due to impaired CSF clearance, indicating a disrupted glymphatic system [21]. Previous research has also linked CP volume to cognitive decline and cerebrovascular damage, reinforcing the idea that CP enlargement was closely related to SVD pathophysiology through its influence on CSF dynamics [22].

Our findings demonstrate a marked correlation between retinal vascular metrics and SVD burden, as well as its imaging markers, consistent with previous studies. Reduced arterial FD has been associated with deep WMH scores in patients with minor ischemic stroke [23], and decreased VD in specific retinal regions correlates markedly with SVD burden and severe WMH [8]. Measures such as FD and VD reflect the complexity of the retinal vascular network; reductions in these measures may indicate vascular rarefaction or remodeling due to chronic ischemic damage in both the brain and eyes [24]. Although Ikram et al. [25] did not find a direct association between venular dilation and SVD burden in the Rotterdam Scan Study, they reported a link between venular dilation and marked SVD progression, possibly due to relatively mild white matter damage at baseline. This suggests that venular dilation may serve as a compensatory mechanism in response to increased intracranial pressure or impaired venous drainage, contributing to vascular stress without directly affecting specific markers of white matter damage.

We found that PSMD correlated with retinal vascular metrics comparable to those linked with WMH, but with higher significance. As a novel biomarker for SVD, this study is the first to investigate relationship of PSMD with retinal imaging metrics, extending previous findings that PSMD is markedly correlated with WMH and consistently outperforms conventional imaging biomarkers in explaining variance in cognitive processing speed [17]. This strengthens the evidence for a shared vascular injury mechanism between the eye and brain, suggesting that SVD may have a systemic impact on both organs.

CP plays a vital role in CSF production and immune surveillance, making it a key player in neuroinflammatory processes. Previous studies have shown CP enlargement in patients with optic neuritis, indicating its involvement in such conditions [26]. Our study presents an innovative finding by identifying a marked correlation between increased CP volume and reduced FD and VD of retina, suggesting a new link between CP dysfunction and retinal vascular changes. To the best of our knowledge, this is the first in vivo imaging study in humans that demonstrates the connection between the CP and retinal blood vessels, although previous research has proposed the hypothesis that CSF flows into the optic nerve and optic disc along the perivascular spaces of the central retinal vessels in a microgravity environment [27]. A potential mechanism for this association may involve impaired CSF clearance or disruption of the blood–CSF barrier, leading to the accumulation of neurotoxic substances, neurovascular inflammation, and endothelial dysfunction, which in turns negatively impact retinal vascular integrity [28–30].

Through structural equation modeling, we confirmed that retinal vascular morphology was almost equally influenced by both cerebrovascular injury and CP dysfunction in SVD patients. Each independent pathway aligned with our previous results from traditional statistical analysis. Importantly, we found that neither factor fully substitutes for the other, highlighting the distinct and complementary roles of CSF dysfunction and brain vascular damage in shaping retinal vascular changes.

Using retinal imaging to predict brain changes has become an emerging area of research. Zee et al. [15] utilized retinal images and extracted vascular features to estimate the probability of age-related WMH in specific brain regions. Ji et al. [31] incorporated patient demographic data with fundus photography and optical coherence tomography to predict SVD diagnosis, achieving AUC values of 0.72 and 0.73, respectively. Our study used 5 retinal vascular markers and basic demographic data to consistently achieve higher accuracy in predicting not only severe SVD burden (AUC = 0.82 ± 0.07) but also the upper quartiles of WMH volumes (0.77 ± 0.01), PSMD values (0.81 ± 0.06), and CP volumes (0.80 ± 0.03). By additionally utilizing the UKB dataset, which includes participants from diverse ethnic backgrounds, we demonstrated the reproducibility of the selected 5 retinal imaging biomarkers for predicting both WMH and CP volumes across a heterogeneous population. In the UKB cohort, the WMH prediction model achieved an AUC of 0.76 ± 0.01, closely aligning with the META-KLS (MEdical imaging sTudy bAsed on KaiLuan Study) cohort’s 0.77 ± 0.01, while the CP volume prediction model showed an AUC of 0.81 ± 0.01, consistent with the 0.80 ± 0.03 in META-KLS. This consistency across datasets underscores the robustness and reliability of the model’s predictive performance. In conclusion, external validation using the UKB dataset further confirmed the lightweight retinal vessel-based model’s effectiveness in predicting neuroimaging markers associated with SVD. The prediction models in this study suggest the potential of retinal imaging to clinically predict not only cerebral vascular impairment but also disruptions in CSF clearance.

There are still several limitations in this study. First, the SVD score, derived from Wardlaw’s scale, offers a comprehensive assessment of overall SVD-related damage. While specific imaging markers such as PSMD and WMH were employed to assess white matter microstructural integrity and microvascular injury, the inherent heterogeneity among participants underscores the need for future studies to specifically investigate the associations between ocular and cerebral parameters across distinct SVD imaging characteristics. Second, we selected 5 retinal imaging features from an initial set of 30 based on their correlation with SVD burden. While these features were carefully chosen to ensure a robust analysis, the relationship may vary depending on specific brain markers. To improve the accuracy of future prediction models, customizing the selection of retinal markers to specific brain imaging biomarkers could be considered. Third, age and sex distribution showed marked differences across different SVD burden scores, which can be attributed to the underlying mechanisms of SVD and the fact that the original dataset was not filtered. Future studies should include more diverse ethnicities to enhance generalizability.

In conclusion, changes in retinal vascular morphology are influenced by both cerebrovascular injury and CP dysfunction. Retinal markers can effectively predict SVD burden, and SVD-related imaging markers such as WMH and PSMD, and CP volume. These findings offer new imaging evidence of the connection between the eye and brain, underscoring the potential of retinal imaging as a noninvasive tool for large-scale SVD screening and diagnosis.

## Materials and Methods

### Study design and participants

The participants of this study were from META-KLS at Hebei, China, details of which were previously depicted by Sun et al. [32]. In brief, META-KLS is a prospective, community-based cohort study, which recruited primarily the employees of Kailuan (Group) Co. Ltd. and local residents. Multi-modality imaging examinations were performed to assess the association of imaging phenotypes between eye and brain in the preclinical diseases. A total of 1,114 participants from December 2020 to August 2022 who performed at least one modal MRI scan was included, and those with unqualified clinical assessment and incomplete MRI or fundus photography data were excluded. This study follows the Strengthening the Reporting of Observational Studies in Epidemiology initiative [33] and has been approved by the Medical Ethics Committee of the Kailuan General Hospital. Written informed consent was acquired from each participant.

### Clinical measurement

The clinical measurement was conducted via face-to-face survey and examination at KaiLuan Hospital by trained researchers. Demographic characteristics (age, sex, and years of education) and vascular risk factors (hypertension, diabetes, dyslipidemia, BMI, and drinking and smoking status) were collected. Cognitive function was evaluated using the Beijing version of the Montreal Cognitive Assessment scale with a maximum score of 30 points and 7 cognitive domains [34].

### Imaging acquisition

Brain MRI examinations were performed at 3.0-T scanner with an 8-channel phase-array coil (MR750w; GE, USA) following a standardized protocol. The protocol included a 3-dimensional brain volume for high-resolution T1-weighted imaging [repetition time (TR)/echo time (TE) = 6.7/2.6 [ms], flip angle = 15°, field of view = 25.6 × 25.6 cm^2^], which was utilized to analyze CP volume. Diffusion tensor imaging (8,000/97.9 [ms], 90°, 24 × 24 cm^2^, 15 directions, *b* = 1,000 mm^2^/s) was employed to calculate PSMD value. Three-dimensional fluid-attenuated inversion recovery imaging (FLAIR; 5,000/1,147 [ms], 90°, 25.6 × 25.6 cm^2^) was used for the automated quantification of WMH volume, as well as manual evaluation of WMH and lacune scores. Two-dimensional T2-weighted imaging (5,842/103 [ms], 142°, 24 × 24 cm^2^) was acquired for manual assessment of perivascular spaces. Susceptibility-weighted imaging (39.7/23.9 [ms], 16°, 24 × 24 cm^2^) was performed for the manual evaluation of cerebral microbleeds. Bilaterally macula and optic disc-centered retinal fundus photographs (imaging size, 2,576 × 1,934 pixels) were captured by a Topcon Deep Range Imaging OCT Triton Device (Triton; Topcon, Japan).

### Assessment of cerebral SVD

SVD burden scores were evaluated on a scale of 0 to 4 based on Wardlaw’s scale [35] by experienced radiologists with 12 years of expertise in brain MRI, with any discrepancies resolved through consensus or adjudication by a senior radiologist possessing over 15 years of experience. Points were assigned as follows: 1 point for the presence of lacunes, defined as one or more lacunes, and 1 point for any cerebral microbleeds. Enlarged perivascular spaces in the basal ganglia were assigned 1 point if graded as moderate to severe (grades 2 to 4, *N* > 10). WMH received 1 point if characterized by either confluent deep WMH (Fazekas score 2 or 3) or irregular periventricular WMH extending into the deep white matter (Fazekas score 3).

### Cerebral imaging marker analysis

The CP, responsible for approximately 80% of the daily CSF production [36], was segmented by FastSurfer (version 2.0.4), a fully convolutional neural network trained on extensive manually labeled datasets to segment brain structures. The CP volume was calculated as a biomarker for CSF dynamics, with an enlargement indicating potential dysfunction in CSF circulation [37].

The WMH volume for each participant was computed using the “Lesion Prediction Algorithm” [38], an unsupervised method within the Lesion Segmentation Toolbox of the SPM12 software package [39]. This algorithm uses a probabilistic model to detect and segment lesions in the brain by leveraging both FLAIR images and the underlying tissue probabilities. After segmentation, the total WMH volume was calculated by summing the volumes of the identified lesions across all brain regions.

The PSMD value was automatically calculated following a well-established protocol [17] using FSL (version 6.0). In brief, PSMD is derived based on skeletonized mean diffusivity from diffusion tensor imaging and serves as a reliable indicator of SVD severity [17]. Higher PSMD values generally correspond to more severe SVD.

All analysis results were subjected to manual quality control to ensure the absence of errors in the analysis.

### Ocular imaging marker analysis

FD quantifies the complexity of retinal vascular branching, with higher values reflecting a more intricate network structure. VD measures the proportion of the retinal area occupied by blood vessels, indicating the extent of vascularization. VAW represents the mean caliber of venules, providing insight into venous vessel characteristics. Additionally, arteriolar FD captures the complexity of the arteriolar network, while arteriolar vessel density quantifies the density of arterioles in the retina. These biomarkers were extracted from fundus photography using AutoMorph [40], following steps that included background removal, image quality grading to exclude poor images, anatomical segmentation of arterioles and venules, and measurement of vascular morphology feature. The segmentation results of retinal vessels were reviewed by experienced analysts to ensure accuracy and reliability. All biomarkers were analyzed within the annulus 0.5 to 2 optic disc diameter from the disc margin in optic disc-centered images of each participant’s right eye.

### Prediction models

We developed support vector machine models to evaluate the predictive ability of retinal imaging markers for cerebrovascular damage, CP enlargement, and SVD burden. All participants were included in the analysis, with those having WMH volumes in the upper quartile or higher classified as the target group and marked as 1, while the remaining participants were classified as nontargets and marked as 0. A similar approach was applied for PSMD and CP volume. For SVD burden prediction, participants with a score of 3 or 4 were classified as the target group and marked as 1. Input features included 5 retinal biomarkers (FD, VD, arterial FD, arterial VD, and VAW) and demographic variables (age, sex, and education). Feature scaling was applied using StandardScaler to standardize the data to a mean of 0 and a standard deviation of 1, enhancing the performance of the machine learning models. Considering the class imbalance in the dataset, RandomOverSampler was employed to balance the training samples by oversampling the minority class. The training and evaluation were performed using a 5-fold stratified cross-validation strategy to ensure that both classes were proportionately represented in each fold. Linear kernel support vector machine models were implemented to predict the targets. The models were trained on the training data in each fold and evaluated on the corresponding test set. Key performance metrics included accuracy, AUC value, sensitivity, and specificity, which were calculated for each fold. Confusion matrices were generated to derive sensitivity and specificity, ensuring a comprehensive assessment of the model’s ability to correctly classify both classes.

### External validation

To confirm the consistency of the prediction model based on vascular and CSF connections across datasets and validate its external reliability, we further used UKB dataset, which is a large, prospective cohort study that recruited participants from 22 centers across the UK between 2006 and 2010 [41]. All participants were invited for imaging and underwent screening to assess safety and tolerance. Detailed information on the study’s design, recruitment, and baseline assessments were described previously [41].

Cerebral imaging was performed on the 3T Siemens Skyra MRI scanner, and fundus photographs (45° field of view, centered to macula) were captured using a digital Topcon-1000 integrated ophthalmic camera (Topcon 3D OCT1000 Mark II, Topcon Corp., Tokyo, Japan).

Considering the participants who underwent both eye and brain imaging and excluding those with low-quality images unsuitable for analysis, a total of 5,691 participants were included. WMH volume (field identifier 25781) and total CP volume of left (field identifier 26567) and right (field identifier 26598) hemispheres were obtained from the UKB-provided fields. WMH was segmented using the Brain Intensity AbNormality Classification Algorithm tool [42] on FLAIR imaging, and CP was segmented using FreeSurfer [43] on 3-dimensional T1-weighted imaging. The full acquisition protocols are provided elsewhere [44], and those included in this analysis are as follows: FLAIR imaging (resolution = 1.05 × 1 × 1 mm^3^, field of view = 192 × 256 × 256 matrix), and T1-weighted imaging (resolution = 1 × 1 × 1 mm^3^, field of view = 192 × 256 × 256 matrix, TR/TE = 2,000/2.01 [ms]). The fundus photograph of each participant’s right eye was directly obtained from the UKB database (field identifier 21016). Ocular imaging markers were analyzed using the same methodology applied to the META-KLS dataset and are available online and are available online (see details in Data Availability).

### Statistical analysis

Statistical analysis was performed using SPSS (version 26.0; IBM, Armonk, NY, USA). For descriptive statistics, we used the χ^2^ test for categorical variables and one-way analysis of variance (ANOVA) or Kruskal–Wallis test for continuous variables. A generalized linear model was used to assess differences in cerebral and retinal imaging markers across varying levels of SVD burden, with estimated marginal mean difference representing the differences between higher burden levels (1, 2, and ≥3) and level 0. Retinal imaging biomarkers with a *P* value for trend less than 0.01 were selected in this study. Age, sex, and education were controlled for in model 1, and hypertension, diabetes, dyslipidemia, BMI, current smoking, and drinking status were added to model 2. Pearson’s bivariate correlation analysis was used to examine the relationship between cerebral and retinal imaging biomarkers, with significance defined as a 2-tailed *P* < 0.05.

Partial least squares structural equation modeling (SmartPLS, version 3.2.9) [45] was implemented to assess the contribution of CP enlargement and cerebrovascular damage (indicated by increased WMH volume or PSMD value) to changes in retinal vascular morphology. Two separate models were constructed: one incorporating CP volumes and WMH volumes, and the other incorporating CP volumes and PSMD values. Retinal vascular morphology was set as the latent variable, formed by the observed variables that were selected according to the significant correlation with SVD burden (*P* < 0.01). The standardized root mean square residual measures the difference between observed and predicted correlations, with a value of less than 0.08 indicating a well-fitting model [46]. Outer weight (OW) indicates the relative contribution of each indicator to its corresponding latent variable, with higher absolute values indicating a stronger contribution to the latent variable.

## Data Availability

This study was registered on clinicaltrials.gov (no. NCT05453877).Ocular imaging markers of UKB are available online (https://github.com/Winniework/Retina-CSVD). The META-KLS data are available from the corresponding author on reasonable request.

## References

[1] Zhu H-H, Li S-S, Wang Y-C, Song B, Gao Y, Xu Y-M, Li Y-S. Clearance dysfunction of trans-barrier transport and lymphatic drainage in cerebral small vessel disease: Review and prospect. Neurobiol Dis. 2023;189, Article 106347. 10.1016/j.nbd.2023.106347 37951367

[2] Pantoni L. Cerebral small vessel disease: From pathogenesis and clinical characteristics to therapeutic challenges. Lancet Neurol. 2010;9(7):689–701. 20610345 10.1016/S1474-4422(10)70104-6

[3] De Leeuw F, de Groot JC, Achten E, Oudkerk M, Ramos LM, Heijboer R, Hofman A, Jolles J, van Gijn J, Breteler MM. Prevalence of cerebral white matter lesions in elderly people: A population based magnetic resonance imaging study. The Rotterdam Scan Study. J Neurol Neurosurg Psychiatry. 2001;70(1):9–14. 11118240 10.1136/jnnp.70.1.9 PMC1763449

[4] Pasi M, Cordonnier C. Clinical relevance of cerebral small vessel diseases. Stroke. 2020;51(1):47–53. 31752613 10.1161/STROKEAHA.119.024148

[5] Zhang J, Gu Y, Chen A, Yu Y. Unveiling dynamic system strategies for multisensory processing: From neuronal fixed-criterion integration to population Bayesian inference. Research. 2022;2022:9787040. 36072271 10.34133/2022/9787040 PMC9422331

[6] Cabrera DeBuc D, Somfai GM, Koller A. Retinal microvascular network alterations: Potential biomarkers of cerebrovascular and neural diseases. Am J Phys Heart Circ Phys. 2017;312(2):H201–H212. 10.1152/ajpheart.00201.2016 PMC5336575 27923786

[7] Delaey C, Van de Voorde J. Regulatory mechanisms in the retinal and choroidal circulation. Ophthalmic Res. 2000;32(6):249–256. 11015035 10.1159/000055622

[8] Ma L, Wang M, Chen H, Qu Y, Yang L, Wang Y. Association between retinal vessel density and neuroimaging features and cognitive impairment in cerebral small vessel disease. Clin Neurol Neurosurg. 2022;221: Article 107407. 35933965 10.1016/j.clineuro.2022.107407

[9] Mathieu E, Gupta N, Ahari A, Zhou X, Hanna J, Yücel YH. Evidence for cerebrospinal fluid entry into the optic nerve via a glymphatic pathway. Invest Ophthalmol Vis Sci. 2017;58(11):4784–4791. 28973323 10.1167/iovs.17-22290

[10] Wostyn P, Van Dam D, Audenaert K, Killer HE, De Deyn PP, De Groot V. A new glaucoma hypothesis: A role of glymphatic system dysfunction. Fluids Barriers CNS. 2015;12:16. 26118970 10.1186/s12987-015-0012-z PMC4485867

[11] Johanson CE. Choroid plexus–cerebrospinal fluid circulatory dynamics: Impact on brain growth, metabolism, and repair. *Neurosci Med*. 2008; pp. 173–200.

[12] Li Y, Zhou Y, Zhong W, Zhu X, Chen Y, Zhang K, He Y, Luo Z, Ran W, Sun J, et al. Choroid plexus enlargement exacerbates white matter hyperintensity growth through glymphatic impairment. Ann Neurol. 2023;94(1):182–195. 36971336 10.1002/ana.26648

[13] Choi JD, Moon Y, Kim H-J, Yim Y, Lee S, Moon W-J. Choroid plexus volume and permeability at brain MRI within the Alzheimer disease clinical spectrum. Radiology. 2022;304(3):635–645. 35579521 10.1148/radiol.212400

[14] Lau AY, Mok V, Lee J, Fan Y, Zeng J, Lam B, Wong A, Kwok C, Lai M, Zee B. Retinal image analytics detects white matter hyperintensities in healthy adults. Ann Clin Transl Neurol. 2019;6(1):98–105. 30656187 10.1002/acn3.688 PMC6331948

[15] Zee B, Wong Y, Lee J, Fan Y, Zeng J, Lam B, Wong A, Shi L, Lee A, Kwok C, et al. Machine-learning method for localization of cerebral white matter hyperintensities in healthy adults based on retinal images. Brain Commun. 2021;3(3):fcab124. 34222872 10.1093/braincomms/fcab124 PMC8249101

[16] Wardlaw JM, Smith C, Dichgans M. Small vessel disease: Mechanisms and clinical implications. Lancet Neurol. 2019;18(7):684–696. 31097385 10.1016/S1474-4422(19)30079-1

[17] Baykara E, Gesierich B, Adam R, Tuladhar AM, Biesbroek JM, Koek HL, Ropele S, Jouvent E, Alzheimer’s Disease Neuroimaging Initiative, Chabriat H, et al. A novel imaging marker for small vessel disease based on skeletonization of white matter tracts and diffusion histograms. Ann Neurol. 2016;80(4):581–592. 27518166 10.1002/ana.24758

[18] Nylander R, Fahlström M, Rostrup E, Kullberg J, Damangir S, Ahlström H, Lind L, Larsson E-M. Quantitative and qualitative MRI evaluation of cerebral small vessel disease in an elderly population: A longitudinal study. Acta Radiol. 2018;59(5):612–618. 28814098 10.1177/0284185117727567

[19] Iliff JJ, Wang M, Liao Y, Plogg BA, Peng W, Gundersen GA, Benveniste H, Vates GE, Deane R, Goldman SA, et al. A paravascular pathway facilitates CSF flow through the brain parenchyma and the clearance of interstitial solutes, including amyloid β. Sci Transl Med. 2012;4(147):147ra111. 10.1126/scitranslmed.3003748 PMC3551275 22896675

[20] Mestre H, Mori Y, Nedergaard M. The brain’s glymphatic system: Current controversies. Trends Neurosci. 2020;43(7):458–466. 32423764 10.1016/j.tins.2020.04.003 PMC7331945

[21] Damkier HH, Brown PD, Praetorius J. Cerebrospinal fluid secretion by the choroid plexus. Physiol Rev. 2013;93(4):1847–1892. 24137023 10.1152/physrev.00004.2013

[22] Song L, Li Y, Han X, Wang J, Li C, Cong L, Hou T, Wang Y, Du Y, Qiu C. Choroid plexus volume, cognitive spectrum, and biomarkers of brain aging in older adults: The MIND-China MRI Study. Alzheimers Dement. 2023;19(S17): Article e073068.

[23] McGrory S, Ballerini L, Doubal FN, Staals J, Allerhand M, Valdes-Hernandez MDC, Wang X, MacGillivray T, Doney ASF, Dhillon B, et al. Retinal microvasculature and cerebral small vessel disease in the Lothian Birth Cohort 1936 and Mild Stroke Study. Sci Rep. 2019;9(1):6320. 31004095 10.1038/s41598-019-42534-x PMC6474900

[24] Nadal J, Deverdun J, de Champfleur NM, Carriere I, Creuzot-Garcher C, Delcourt C, Chiquet C, Kawasaki R, Villain M, Ritchie K, et al. Retinal vascular fractal dimension and cerebral blood flow, a pilot study. Acta Ophthalmol. 2020;98(1):e63–e71. 31545560 10.1111/aos.14232

[25] Ikram MK, De Jong FJ, Van Dijk EJ, Prins ND, Hofman A, Breteler MMB, De Jong PTVM. Retinal vessel diameters and cerebral small vessel disease: The Rotterdam Scan Study. Brain. 2005;129(Pt 1):182–188. 16317022 10.1093/brain/awh688

[26] Klistorner S, Van der Walt A, Barnett MH, Butzkueven H, Kolbe S, Parratt J, Yiannikas C, Klistorner A. Choroid plexus volume is enlarged in clinically isolated syndrome patients with optic neuritis. Mult Scler J. 2023;29(4-5):540–548. 10.1177/13524585231157206 36876595

[27] Wostyn P, Mader TH, Gibson CR, Killer HE. The escape of retrobulbar cerebrospinal fluid in the astronaut’s eye: Mission impossible? Eye. 2019;33(10):1519–1524. 31065103 10.1038/s41433-019-0453-8 PMC7002684

[28] Simon MJ, Iliff JJ. Regulation of cerebrospinal fluid (CSF) flow in neurodegenerative, neurovascular and neuroinflammatory disease. Biochim Biophys Acta. 2016;1862(3):442–451. 26499397 10.1016/j.bbadis.2015.10.014 PMC4755861

[29] Keep RF, Jones HC, Hamilton MG, Drewes LR. A year in review: Brain barriers and brain fluids research in 2022. Fluids Barriers CNS. 2023;20(1):30. 37085841 10.1186/s12987-023-00429-0 PMC10120509

[30] Liu R, Jia W, Wang Y, Hu C, Yu W, Huang Y, Wang L, Gao H. Glymphatic system and subsidiary pathways drive nanoparticles away from the brain. Research. 2022;2022:9847612. 35360646 10.34133/2022/9847612 PMC8943630

[31] Ji C, Li J, Du C, Lv B, Wu N, Li H, Li R, Hui Y, Xie G, Wu S, et al. Predicting cerebral small vessel disease through retinal scans and demographic data with Bayesian feature selection. Paper presented at: SPIE Medical Imaging; 2024; San Diego, CA, USA.

[32] Sun J, Hui Y, Li J, Zhao X, Chen Q, Li X, Wu N, Xu M, Liu W, Li R, et al. Protocol for multi-modality MEdical imaging sTudy bAsed on KaiLuan study (META-KLS): Rationale, design and database building. BMJ Open. 2023;13(2): Article e067283. 10.1136/bmjopen-2022-067283 PMC9923283 36764715

[33] Von Elm E, Altman DG, Egger M, Pocock SJ, Gøtzsche PC, Vandenbroucke JP, Strobe Initiative. The Strengthening the Reporting of Observational Studies in Epidemiology (STROBE) statement: Guidelines for reporting observational studies. Lancet. 2007;370(9596):1453–1457. 18064739 10.1016/S0140-6736(07)61602-X

[34] Yu J, Li J, Huang X. The Beijing version of the Montreal Cognitive Assessment as a brief screening tool for mild cognitive impairment: A community-based study. BMC Psychiatry. 2012;12:156. 23009126 10.1186/1471-244X-12-156 PMC3499377

[35] Staals J, Makin SD, Doubal FN, Dennis MS, Wardlaw JM. Stroke subtype, vascular risk factors, and total MRI brain small-vessel disease burden. Neurology. 2014;83(14):1228–1234. 25165388 10.1212/WNL.0000000000000837 PMC4180484

[36] Damkier H, Praetorius J. Structure of the mammalian choroid plexus. In: Praetorius J, Blazer-Yost B, Damkier H, editors. *Role of the choroid plexus in health and disease*. New York (NY): Springer; 2020. p. 1–33.

[37] Tadayon E, Pascual-Leone A, Press D, Santarnecchi E, Alzheimer’s Disease Neuroimaging Initiative. Choroid plexus volume is associated with levels of CSF proteins: Relevance for Alzheimer’s and Parkinson’s disease. Neurobiol Aging. 2020;89:108–117. 32107064 10.1016/j.neurobiolaging.2020.01.005 PMC9094632

[38] Sun J, Wang L, Gao Y, Hui Y, Chen S, Wu S, Wang Z, Jiang J, Lv H. Discovery of high-risk clinical factors that accelerate brain aging in adults: A population-based machine learning study. Research. 2024;7:0500. 39434838 10.34133/research.0500 PMC11491671

[39] Schmidt P. Bayesian inference for structured additive regression models for large-scale problems with applications to medical imaging [dissertation]. [Munich (Germany)]: LMU Munich; 2017.

[40] Zhou Y, Wagner SK, Chia MA, Zhao A, Woodward-Court P, Xu M, Struyven R, Alexander DC, Keane PA. AutoMorph: Automated retinal vascular morphology quantification via a deep learning pipeline. Transl Vis Sci Technol. 2022;11(7):12. 10.1167/tvst.11.7.12 PMC9290317 35833885

[41] Sudlow C, Gallacher J, Allen N, Beral V, Burton P, Danesh J, Downey P, Elliott P, Green J, Landray M, et al. UK biobank: An open access resource for identifying the causes of a wide range of complex diseases of middle and old age. PLOS Med. 2015;12(3): Article e1001779. 25826379 10.1371/journal.pmed.1001779 PMC4380465

[42] Griffanti L, Zamboni G, Khan A, Li L, Bonifacio G, Sundaresan V, Schulz UG, Kuker W, Battaglini M, Rothwell PM, et al. BIANCA (Brain Intensity AbNormality Classification Algorithm): A new tool for automated segmentation of white matter hyperintensities. NeuroImage. 2016;141:191–205. 27402600 10.1016/j.neuroimage.2016.07.018 PMC5035138

[43] Fischl B. FreeSurfer. NeuroImage. 2012;62(2):774–781. 22248573 10.1016/j.neuroimage.2012.01.021 PMC3685476

[44] Alfaro-Almagro F, Jenkinson M, Bangerter NK, Andersson JLR, Griffanti L, Douaud G, Sotiropoulos SN, Jbabdi S, Hernandez-Fernandez M, Vallee E, et al. Image processing and Quality Control for the first 10,000 brain imaging datasets from UK Biobank. NeuroImage. 2018;166:400-24. 29079522 10.1016/j.neuroimage.2017.10.034 PMC5770339

[45] Hair JF, Risher JJ, Sarstedt M, Ringle CM. When to use and how to report the results of PLS-SEM. Eur Bus Rev. 2019;31(1):2–24.

[46] Lt H, Bentler PM. Cutoff criteria for fit indexes in covariance structure analysis: Conventional criteria versus new alternatives. Struct Equ Model Multidiscip J. 1999;6(1):1–55.

