# Supplementary material for: Retinal Vascular Morphology Reflects and Predicts Cerebral Small Vessel Disease: Evidences from Eye–Brain Imaging Analysis
Source: Research (Wash D C). 2025 Mar 6;8:0633. doi: 10.34133/research.0633 (PMC11883085; doi:10.34133/research.0633)
Supplement: Supplementary 1 — Figs. S1 and S2 [file research.0633.f1.docx]

SUPPLEMENTARY MATERIALS


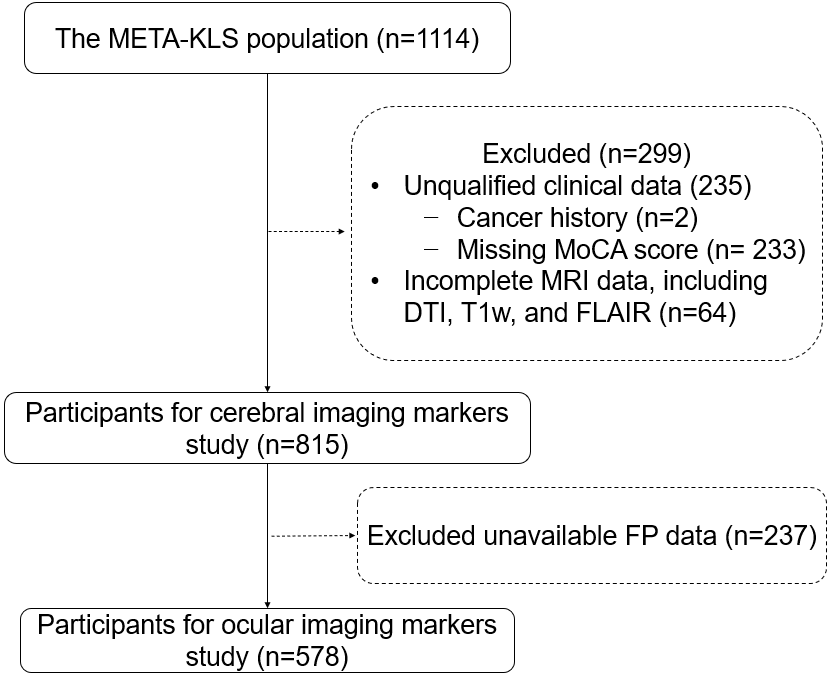


**Figure S1. Flowchart of participants selection.**

Abbreviations: CBF, cerebral blood flow; DTI, diffusion tensor imaging; FLAIR, fluid-attenuated inversion recovery; FP, fundus photography; META-KLS, Multi-modality MEdical imaging sTudy bAsed on KaiLuan Study; MoCA, Montreal Cognitive Assessment; MRI, magnetic resonance imaging; T1w, T1-weighed.


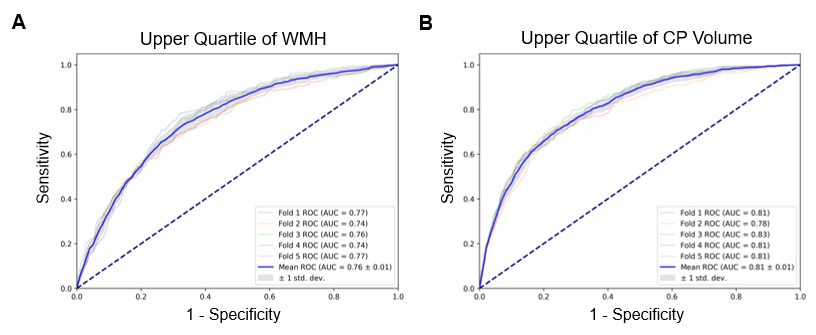


**Figure S2. Receiver operating characteristic curves of validation models for predicting upper quartile of WMH (A) and CP volume (B).**

Abbreviations: AUC, area under the curve; CP, choroid plexus; ROC, receiver operating characteristic; WMH, white matter hyperintensities.
